# Similarity index for intuitive assessment of three-dimensional facial asymmetry

**DOI:** 10.1038/s41598-019-47477-x

**Published:** 2019-07-29

**Authors:** Sun Mi Kwon, Jae Joon Hwang, Yun-Hoa Jung, Bong-Hae Cho, Kee-Joon Lee, Chung-Ju Hwang, Sung-Hwan Choi

**Affiliations:** 1 Department of Orthodontics, Institute of Craniofacial Deformity, Yonsei University College of Dentistry, Seoul, Republic of Korea; 2 Department of Oral and Maxillofacial Radiology, Institute of Translational Dental Sciences, School of Dentistry, Pusan National University, Yangsan, Republic of Korea

**Keywords:** Translational research, Outcomes research

## Abstract

Evaluation of facial asymmetry generally involves landmark-based analyses that cannot intuitively assess differences in three-dimensional (3D) stereoscopic structures between deviation and non-deviation sides. This study tested a newly developed similarity index that uses a mirroring technique to intuitively evaluate 3D mandibular asymmetry, and characterised the resulting lower facial soft tissue asymmetry. The similarity index was used to evaluate asymmetry before and after surgery in 46 adult patients (27 men, 19 women; age, 22 ± 4.8 years) with skeletal Class III malocclusion and facial asymmetry who underwent conventional bimaxillary orthognathic surgery. Relative to the midsagittal plane used as the reference plane, the non-overlapping volume of the mandible significantly decreased, and the similarity index significantly increased after surgery. Similarity indexes of the mandible and lower facial soft tissue were strongly negatively correlated with non-overlapping volumes of each measurement. Differences in bilateral hemi-mandibular and hemi-lower facial soft tissue surface and volume measurements before surgery were significantly negatively correlated with similarity indexes of the mandible before and after surgery. This newly developed similarity index and non-overlapping volume using a mirroring technique can easily and intuitively evaluate overall 3D morphological discrepancies, especially 3D mandibular asymmetry, before and after surgery in skeletal Class III patients with facial asymmetry.

## Introduction

Facial asymmetry is a primary complaint of adult patients who desire orthognathic surgery. The major cause of facial asymmetry is known as bilateral asymmetry of the mandible and is accompanied by facial asymmetry in approximately 50% of patients with mandibular prognathism^1–3^. Thus far, evaluations of mandibular asymmetry have included division of the mandible into deviation and non-deviation sides, primarily based on the direction of the menton deviation; using cephalometric landmarks, the differences in linear and angular measurements of bilateral mandibular rami or bodies can then be compared^4–7^. However, such landmark-based analysis cannot evaluate the mandibular asymmetry of the entire structure because mandibular asymmetry can arise from bilateral three-dimensional (3D) morphological discrepancies other than positional differences on the coordinates of the landmarks^8^. Further 3D stereoscopic evaluation using surface area or volumetric measurements, rather than two-dimensional (2D) measurements, may be advantageous to intuitively judge the degree of mandibular asymmetry before surgery, as well as the outcome after surgery, for each asymmetric patient with ramus or body origin^9,10^.

Although lower facial soft tissue is important in evaluation of facial aesthetics and surgical outcomes of facial asymmetry patients, existing methods using 3D laser or optical scan are also dependent on several landmarks on the soft tissue and mainly analyse the surface morphology^5,6^; therefore, it is also insufficient to obtain an intuitive understanding of the extent of bilateral asymmetry or the amount of change after surgery.

Therefore, the aims of this study were to test a newly developed similarity index that uses a mirroring technique to easily and intuitively evaluate 3D mandibular asymmetry; to characterise the resulting lower facial soft tissue asymmetry; and to use this new method and the existing (conventional) method to compare asymmetry before and after surgery in patients with skeletal Class III malocclusion and facial asymmetry who underwent conventional bimaxillary orthognathic surgery.

## Results

### Mandibular and lower facial soft tissue measurements and bilateral differences before and 1 year after surgery

This study included 46 patients (27 men, 19 women; age, 22 ± 4.8 years). Genioplasty was performed in 23 of the patients. Before surgery, the length difference of bilateral ramus and body were statistically significantly different between deviated (Dev) and non-deviated (N-Dev) sides (*P* = 0.001). The length of both ramus and body on the N-Dev side were longer than those on the Dev side by 1.8 (standard deviation; SD, 3.6) mm and 2.6 (SD, 2.6) mm, respectively (Table 1).

**Table 1:** Comparison of mandibular and lower facial soft tissue measurements between deviated (Dev) and non-deviated (N-Dev) sides before (T1) and 1 year after (T2) surgery.

Variable	T1				T2				T1–T2
	Dev	N-Dev	Difference	*P* value^†^	Dev	N-Dev	Difference	*P* value^†^	*P* value^‡^
**Linear measurements (mm)**									
Ramal length	61.4 ± 5.9	63.2 ± 5.5	−1.8 ± 3.6	0.001	57.2 ± 11.1	57.5 ± 11.1	−0.2 ± 12.4	0.873	0.397
Body length	90.6 ± 5.3	93.3 ± 4.5	−2.6 ± 2.6	< 0.001	85.4 ± 4.9	86.8 ± 4.5	−1.3 ± 3.5	0.011	0.026
**Surface area measurements (×10**^**3**^ **mm**^2^**)**									
Hemi-mandibular surface (AMP)	11.0 ± 1.4	11.2 ± 1.3	−0.2 ± 0.5	0.018	10.6 ± 1.4	10.6 ± 1.3	−0.0 ± 0.5	0.807	0.007
Ramal surface	4.1 ± 0.8	4.3 ± 0.8	−0.2 ± 0.7	0.076	4.0 ± 0.7	4.1 ± 0.6	0.1 ± 0.4	0.093	0.328
Body surface	6.8 ± 1.1	6.8 ± 1.0	0.0 ± 0.8	0.998	6.6 ± 0.9	6.5 ± 0.8	0.0 ± 0.6	0.311	0.333
Hemi-mandibular surface (MSP)	11.4 ± 1.4	10.8 ± 1.3	0.6 ± 0.5	< 0.001	10.7 ± 1.3	10.5 ± 1.3	0.2 ± 0.5	0.008	<0.001
**Volumetric measurements (×10**^**3**^ **mm**^3^**)**									
Hemi-mandibular volume (AMP)	40.7 ± 7.6	41.6 ± 8.2	−0.8 ± 2.6	0.035	41.0 ± 8.0	41.5 ± 8.3	−0.4 ± 2.0	0.112	0.125
Ramal volume	11.7 ± 3.5	12.2 ± 3.1	−0.4 ± 2.7	0.288	12.3 ± 3.1	13.0 ± 2.9	−0.6 ± 1.8	0.021	0.471
Body volume	29.0 ± 6.0	29.4 ± 6.6	−0.4 ± 3.1	0.393	28.7 ± 5.6	28.5 ± 6.0	0.1 ± 2.5	0.662	0.111
Hemi-mandibular volume (MSP)	42.4 ± 7.8	39.9 ± 8.1	2.5 ± 2.9	< 0.001	41.6 ± 8.0	41.0 ± 8.2	0.5 ± 2.2	0.086	<0.001
**Soft tissue measurements**									
Hemi-lower facial surface (×10^3^ mm^2^)	49.8 ± 3.5	48.3 ± 3.8	1.5 ± 3.0	0.002	45.3 ± 3.2	44.9 ± 3.0	0.4 ± 2.5	0.211	<0.001
Hemi-lower facial volume (×10^3^ mm^3^)	971.8 ± 136.6	935.1 ± 131.8	36.7 ± 93.1	0.010	908.7 ± 118.9	896.0 ± 117.5	12.6 ± 78.5	0.280	<0.001

AMP, absolute mandibular midsagittal plane; MSP, facial midsagittal plane.

Difference indicates measurements on Dev side minus measurements on N-Dev side.

^†^*P* values were calculated by paired *t*-test.

^‡^Difference between T1 and T2 was calculated by paired *t*-test.

When comparing the surface area and volume of bilateral hemi-mandibles with absolute mandibular midsagittal plane (AMP) as the reference plane, both the surface area (*P* = 0.018) and volume (*P* = 0.035) of the hemi-mandible on the N-Dev side were statistically larger than those on the Dev side, with mean differences of 0.2 (SD, 0.5) × 10^3^ mm^2^ and 0.8 (SD, 2.6) × 10^3^ mm^3^, respectively (Table 1). However, differences in the surface area and volume between bilateral ramus and body segments were not statistically significant.

When using the midsagittal plane (MSP) as the reference plane, both the surface area (*P* < 0.001) and volume (*P* < 0.001) were significantly larger in the Dev side than in the N-Dev side, with mean differences of 0.6 (SD, 0.5) × 10^3^ mm^2^ and 2.5 (SD, 2.9) × 10^3^ mm^3^, respectively (Table 1). The surface area (*P* = 0.002) and volume (*P* = 0.010) of the hemi-lower facial soft tissue divided by the MSP were significantly larger on the Dev side than on the N-Dev side by 1.5 (SD, 3.0) × 10^3^ mm^2^ and 36.7 (SD, 93.1) × 10^3^ mm^3^ before surgery.

One year after surgery, most measurements did not show significant differences between the Dev and N-Dev sides (Table 1). However, despite significant changes from before (T1) to 1 year after (T2) surgery, the bilateral differences in mandibular body length and hemi-mandibular surface area, based on the MSP, were statistically significant (*P* = 0.011 and *P* = 0.008). Additionally, bilateral ramal volumes, which were not significantly different before surgery, were significantly different at 1 year after surgery (*P* = 0.021).

### Non-overlapping volume and similarity index by mirroring technique

When mirroring and overlapping were performed with AMP as the reference plane, the non-overlapping volume of the mandible, which was 46.4 (SD, 17.5) × 10^3^ mm^3^ before surgery, decreased to 36.1 (SD, 17.4) × 10^3^ mm^3^ one year after surgery; this change was statistically significant (*P* = 0.003) (Table 2). The similarity index also significantly increased from 0.2 (SD, 0.1) to 0.4 (SD, 0.2, *P* = 0.002).

**Table 2:** Non-overlapping volume and similarity index using mirroring technique before (T1) and 1 year after (T2) surgery.

	T1	T2	*P* value^†^
**Mandible**			
Non-overlapping volume (×10^3^ mm^3^) (AMP)	46.4 ± 17.5	36.1 ± 17.4	0.003
Ramus	18.7 ± 6.4 (41.3%)	16.2 ± 7.5 (46.0%)	
Body	27.7 ± 12.0 (58.6%)	19.9 ± 10.7 (53.9%)	
Similarity index (AMP)	0.2 ± 0.1	0.4 ± 0.2	0.002
Non-overlapping volume (×10^3^ mm^3^) (MSP)	33.1 ± 16.1	24.2 ± 11.3	<0.001
Ramus	10.8 ± 5.4 (34.4%)	9.9 ± 4.6 (42.0%)	
Body	22.2 ± 12.9 (65.5%)	14.2 ± 8.0 (57.9%)	
Similarity index (MSP)	0.4 ± 0.1	0.5 ± 0.1	<0.001
**Lower facial soft tissue**			
Non-overlapping volume (×10^3^ mm^3^)	154.6 ± 59.0	138.1 ± 50.2	0.079
Similarity index	0.8 ± 0.0	0.8 ± 0.0	0.385

AMP, absolute mandibular midsagittal plane; MSP, facial midsagittal plane.

^†^Difference between T1 and T2 was calculated by paired *t*-test.

When MSP was used as the reference plane, the non-overlapping volume of the mandible significantly decreased from 33.1 (SD, 16.1) × 10^3^ mm^3^ to 24.1 (SD, 11.3) × 10^3^ mm^3^ (*P* < 0.001); the similarity index also significantly increased from 0.4 (SD, 0.1) to 0.5 (SD, 0.1) (*P* < 0.001) (Table 2). The non-overlapping volume of the lower facial soft tissue, based on MSP, decreased from 154.6 (SD, 59.0) × 10^3^ mm^3^ before surgery to 138.1 (SD, 50.2) × 10^3^ mm^3^ after surgery; however, this change was not statistically significant. The change in similarity index was also not statistically significant (Table 2).

The distribution of non-overlapping volume after mirroring and superimposition was examined by dividing the hemi-mandible into ramus and body segment at T1 and T2. The proportion of the non-overlapping volume in the body segment was larger than that in the ramal segment after surgery in both cases, using either AMP (53.9% and 46.0%) or MSP (57.9% and 42.0%) as the reference plane; this indicated that the residual asymmetry was larger in the body region than in the ramus region (Table 2).

### Correlations between similarity index and non-overlapping volume, and between similarity index and bilateral measurement difference

Similarity indexes of the mandible and lower facial soft tissue, based on the MSP at each time point, showed strongly negative correlations with the non-overlapping volumes of each measurement at each time point, with correlation coefficients that ranged from −0.948 to −0.902 (*P* < 0.001); this indicated that the similarity index directly reflected the non-overlapping volume (Table 3). The similarity indexes of the mandible and lower facial soft tissue at T2 were significantly negatively correlated with the non-overlapping volumes of the mandible and lower facial soft tissue at T1, respectively. The differences of bilateral hemi-mandibular and hemi-lower facial soft tissue surface and volume measurements at T1 were significantly negatively correlated with the similarity indexes of the mandible at T1 and T2, with correlation coefficients that ranged from −0.316 to −0.692, indicating that the newly proposed similarity index is closely related to the asymmetric severity, especially analysed by conventional methods before surgery (Table 4).

**Table 3:** The correlation coefficient (*P* value) between the similarity index and non-overlapping volume based on the MSP before (T1) and 1 year after (T2) surgery.

Similarity index	T1 non-overlapping volume (mandible)	T1 non-overlapping volume (lower facial soft tissue)	T2 non-overlapping volume (mandible)	T2 non-overlapping volume (lower facial soft tissue)
T1 mandible	−0.902 (<0.001)	NS	−0.526 (<0.001)	NS
T1 lower facial soft tissue	−0.324 (0.028)	−0.948 (<0.001)	NS	NS
T2 mandible	−0.527 (<0.001)	NS	−0.916 (<0.001)	NS
T2 lower facial soft tissue	NS	−0.300 (0.043)	NS	−0.928 (<0.001)

MSP, facial midsagittal plane; NS, not significant.

**Table 4:** The correlation coefficient (*P* value) between the similarity index and difference in bilateral measurements based on the MSP before (T1) and 1 year after (T2) surgery.

Similarity index	T1 hemi-mandibular surface difference	T1 hemi-mandibular volume difference	T1 hemi-lower face surface difference	T1 hemi-lower face volume difference	T2 hemi-mandibular surface difference	T2 hemi-mandibular volume difference	T2 hemi-lower face surface difference	T2 hemi-lower face volume difference
T1 mandible	−0.629 (<0.001)	−0.692 (<0.001)	−0.328 (0.026)	−0.429 (0.003)	NS	−0.486 (0.001)	NS	NS
T1 lower facial soft tissue	−0.404 (0.005)	NS	−0.546 (<0.001)	−0.550 (<0.001)	NS	NS	−0.477 (0.001)	−0.463 (0.001)
T2 mandible	−0.316 (0.032)	−0.452 (0.002)	−0.384 (0.008)	−0.402 (0.006)	−0.356 (0.015)	−0.431 (0.003)	−0.443 (0.002)	−0.416 (0.004)
T2 lower facial soft tissue	NS	NS	NS	NS	NS	NS	−0.518 (<0.001)	−0.466 (0.001)

MSP, facial midsagittal plane; NS, not significant.

## Discussion

The aims of this study were to test a newly developed similarity index that uses a mirroring technique to intuitively evaluate 3D mandibular asymmetry and resulting lower facial soft tissue asymmetry. Interestingly, newly developed similarity indexes of the mandible and lower facial soft tissue directly reflected the non-overlapping volumes of each measurement before and after surgery; in particular, the similarity index of the mandible was significantly negatively correlated with the differences of bilateral hemi-mandibular and hemi-lower facial soft tissue surface and volume measurements before surgery. This means that the similarity index is morphologically easier and more intuitive for use in evaluating the degree of 3D facial asymmetry before and after surgery, compared with the conventional asymmetric evaluation method.

Facial asymmetry can occur due to differences in size or shape of bilateral mandibular structures, as well as positional discrepancy, misalignment, or malrotation of the mandible^11^. Therefore, in this study, we used two reference planes to distinguish between the mandibular asymmetry itself and mandibular misalignment problems. AMP was used as the mandibular internal median plane to evaluate the structural asymmetry of the mandible^12,13^; MSP was used as the clinically applied sagittal reference plane for the evaluation of mandibular asymmetry relative to the face, which is caused by rotation or misalignment, as well as structural asymmetry of the mandible^14^. AMP used in this study is landmark-based, simple to establish, and can be useful for quantitatively comparing bilateral measurement values, such as surface area and volume. However, when mirroring was applied, there were several instances in which the image generated by reflecting the hemi-mandible along the AMP deviated significantly with respect to the existing hemi-mandible of the contralateral side. For example, Table 1 shows that volumetric measurements were significantly different between T1 and T2 in the use of MSP, but not the use of AMP. Mirroring is very sensitive to the reference plane angle, and slight changes in angle can cause considerable errors in the process of overlapping the hemi-mandibles. It is not fully established that the angle formed by the plane passing through the G, B, and Me is appropriate for the mirroring and superimposition process; thus, an alternative plane is needed. Fang *et al*.^15^ proposed a landmark-free voxel-based median plane for dividing the mandible into two symmetrical halves using a computer algorithm, but there is not yet a consensus regarding the standardized median plane^8,16,17^. Therefore, in this study, the relationships between the similarity index and multiple variables were analysed before and after surgery using the MSP, which is applicable to both mandibular and lower facial soft tissue, as a reference plane.

Many previous studies have relied on the conventional method for asymmetry evaluation, which comprises setting a reference plane and determining the positional differences of bilateral landmarks or quantitative differences of bilateral 2D or 3D measurements. However, this method is based on several landmarks that are relatively easy to define; therefore, it encounters difficulty when assessing 3D morphological differences in some areas, such as the lower border of the mandibular body, angle, or parasymphysis. In addition, the method comprising quantitative comparison of bilateral measurements does not reflect differences in the shape or arrangement of the structures, which may occur regardless of similarities in total surface area or bilateral structural volumes. More detailed analysis of the morphological discrepancy of bilateral structures in asymmetric patients is a prerequisite for intuitive understanding of present conditions.

In order to overcome these limitations, several morphological analyses have been attempted with various academic softwares that can manipulate 3D images acquired by cone beam computed tomography (CBCT). Notably, 3D images of a structure can be mirrored, re-registered, and overlapped, and the discrepancies between original and mirrored images can then be visualized through color-coded surface maps^18,19^. In this approach, the degree of discrepancy is expressed as a numerical value representative of the surface distance difference between the two images using the closest point algorithm^20^, modified Hausdorff distances^21^, or root-mean-square difference^22^. However, prior to such assessments, there is a need to determine the actual volume of non-overlapping areas and the ratio of the overlapping volume to the whole volume of the structure; these parameters provide a more immediate understanding of the degree of asymmetry.

In this study, when using the conventional method for determining the degree of asymmetry, differences in the surface area and volume of bilateral structures of the mandible and lower facial soft tissue were no more than 1% of the total surface area and volume of the entire structure before surgery. Conversely, when performing stereoscopic analysis using a mirroring technique, the non-overlapping volume comprises approximately 20–60% of the total mandibular and lower facial soft tissue volumes before surgery. Thus, the difference in shape or arrangement of bilateral structures is much larger than the quantitative difference of the surface area and volume of bilateral structures, thereby contributing to overall asymmetry.

To make this stereoscopic analysis more useful, superimposed hemi-mandibles can be divided into smaller segments to determine the distribution of non-overlapping volumes. In this study, we investigated the non-overlapping volume by dividing the hemi-mandible formed by mirroring and superimposition into two segments, ramus and body, at 1 year after surgery. The results showed that residual asymmetry in the body region was larger than that in the ramus region. This is consistent with the results of Lin *et al*.^19^ who reported that there is a high probability of residual asymmetry in the lower mandibular curvature region of the mandible after orthognathic surgery. Therefore, even if the position of the mandible is modified through repositioning of the maxillomandibular complex to match the facial midline, asymmetry may remain due to differences in the shape or divergence angle of the mandibular body. In this study, the hemi-mandible was only divided into ramus and body segments; however, the non-overlapping volume in angle or parasymphysis areas can also be obtained by establishing alternative sectioning planes.

This stereoscopic analysis can also be applied to adequately assess the asymmetry of the lower facial soft tissue area. However, in the present study, no significant change was found in the non-overlapping volume and similarity index in lower facial soft tissue area before and after surgery. This may be because the surgery improves severe asymmetry of the hard tissue, but soft tissue changes do not directly follow the skeletal changes. In clinical practice, it is difficult to predict changes in soft tissue before and after surgery when planning a treatment plan for orthognathic surgery because changes can be influenced by various factors, such as soft tissue thickness, soft tissue compensation, muscle elongation, mastication preference, sex, and anteroposterior or vertical facial pattern^23–25^. Additionally, unlike the hard tissue, the lower facial soft tissue is not characteristic or distinctive in shape and is generally round; therefore, even if such regions are visually asymmetric, they are less likely than the mandible to exhibit noticeable discrepancy, resulting in a relatively large similarity index. In this regard, further studies of soft tissue asymmetry are needed in which the soft tissue is divided into more detailed regions, concomitant with the use of a larger number of samples. Moreover, in such subsequent studies, it is possible to supplement the soft tissue index accordingly.

This study has limitations in that maxillary regions were excluded from the evaluation. This was because the morphological asymmetry of the maxilla has a minimal effect on the overall facial asymmetry, compared to the mandible; moreover, because the maxilla is attached to the skull, it is difficult to separate from facial structures. In this study, the method of mirroring and superimposition was simply used to evaluate the degree of asymmetry before and after surgery; however, it can also be used in the virtual presurgical simulation to estimate the degree of residual asymmetry and the required amount of augmentation or shaving. Future investigations are needed to apply this method to the virtual presurgical simulation process.

It is difficult to intuitively cope with changes in lower facial soft tissue before and after surgery; although the maxilla was not included, this newly developed similarity index involving a mirroring technique can be used as a more intuitive method to diagnose mandibular asymmetry before surgery, and to analyse residual asymmetry after surgery that can replace landmark-based conventional methods. The newly developed similarity index and non-overlapping volume can reflect differences in shape or arrangement of bilateral structures that contribute to actual asymmetry, rather than quantitative differences in surface area and volume of bilateral structures that are measured using conventional methods. It is expected that this new method can be more effectively applied to actual clinical practice by utilizing the segmentation technique to divide structures using various cutting planes, establish appropriate reference planes, and supplement soft tissue evaluation.

## Methods

### Study design and subjects

This study included 46 adult patients with skeletal Class III malocclusion and facial asymmetry who underwent conventional bimaxillary orthognathic surgery (1-piece Le Fort I and bilateral intraoral vertical osteotomy (IVRO)) with presurgical orthodontic treatment from 2010 to 2017 at Yonsei University Dental Hospital, Seoul, Republic of Korea.

The inclusion criteria for the study were as follows: (1) age older than 18 years; (2) skeletal Class III malocclusion with the angle formed by point A, the nasion, and point B smaller than 0°; (3) menton deviation from the facial MSP >4 mm^14^; and (4) availability of serial CBCT images obtained at T1 and T2 surgery. The exclusion criteria were as follows: (1) history of orthodontic treatment or orthognathic surgery; (2) patients who underwent single-jaw surgery or surgery-first bimaxillary surgery; (3) existing congenital anomalies, such as cleft lip or palate; (4) history of maxillofacial trauma; and (5) inadequate image quality due to motion blurring artefact.

This study was approved by the institutional review board of Yonsei University Dental Hospital (2-2018-0063). This study conformed to the Declaration of Helsinki and was performed in accordance with the current Standards Recommended for the Reporting of Observational Studies in Epidemiology (STROBE). Written informed consent was obtained from all patients before the initiation of treatment.

### Surgical and orthodontic treatment

Before surgery, all patients received pre-surgical orthodontic treatment. After pre-surgical orthodontic treatment, patients underwent conventional bimaxillary surgery, including maxillary Le Fort I osteotomy with posterior impaction and bilateral IVRO for mandibular setback. After 4 weeks of surgery with 2 weeks of intermaxillary fixation^26^, post-surgical orthodontic treatment was performed for 6 months to 1 year.

### Data acquisition, 3D landmark determination, and image reorientation

CBCT data were acquired using Alphard3030 (Alphard Roentgen Ind., Ltd., Kyoto, Japan) at 80 kVp and 10 mA, with 200 mm × 200 mm field of view at T1 and T2. The voxel size was 0.39 mm. The CBCT images were converted to DICOM 3.0 files and stored on a Windows-10 based workstation (Intel Core i7-4770, 32 GB).

Using the medical studio of Invivo 6 (Anatomage, San Jose, CA, USA), fourteen 3D Landmarks were selected in accordance with the procedure used in a previous study (Table 5)^12,13^. The images were reoriented using 2 reference planes: the Frankfort horizontal plane (FHP), passing through the bilateral Po and left Or, and the MSP, passing through the N and S (perpendicular to the FHP). The side of the lateral menton deviation in relation to the MSP was defined as the Dev side and the opposite side was defined as the N-Dev side. After the two reference planes and hard tissue and soft tissue landmarks were configured via Invivo 6 software as described above, the coordinates of 3D landmarks and the binarization thresholds of bone (320-520 HU) and soft tissue (−480–−360 HU) were saved in Excel file format. A custom program using MATLAB 2018a (MathWorks, Natick, MA, USA) was used for successive image processing^27^.

**Table 5:** Descriptions of landmarks used in this study.

Landmark	Definition
N (nasion)	Middle point of nasofrontal suture
S (sella)	Centre of sella turcica
Or (orbitale)	Most inferior point of lower margin of orbit
Po (porion)	Most superior point of external auditory meatus
Con (condylion)	Most superior point of condylar head
J_lat_	Most lateral and deepest point of curvature formed at junction of mandibular ramus and body
J_med_	Most medial and deepest point of curvature formed at junction of mandibular ramus and body
Go_post_ (gonion posterius)	Most posterior point on mandibular angle
Go_mid_ (gonion midpoint)	Midpoint between Go_post_ and Go_inf_ on mandibular angle
Go_inf_ (gonion inferius)	Most inferior point on mandibular angle
Me (menton)	Most inferior midpoint on symphysis
Pog (pogonion)	Most anterior midpoint on symphysis
B (supramentale)	Midpoint of greatest concavity on anterior border of symphysis
G (genial tubercle)	Midpoint on genial tubercle

### Morphological operation of mandible and soft tissue

Separated images of mandible and soft tissue were binarized and morphological operation was performed for surface area and volume calculation. The holes of each binarized image were filled for accurate measurements, based on the method used in a previous study^28^. Morphological closing (a dilation followed by an erosion) was performed for hole filling of the condylar heads, because the upper portion of the condyle has a very thin cortical layer and is likely to be open-ended after binarization. In this article, a sphere structuring element with a radius of 10 pixels was used for morphological closure.

### Measurements

#### Mandibular measurements and bilateral differences

**Figure 1:**
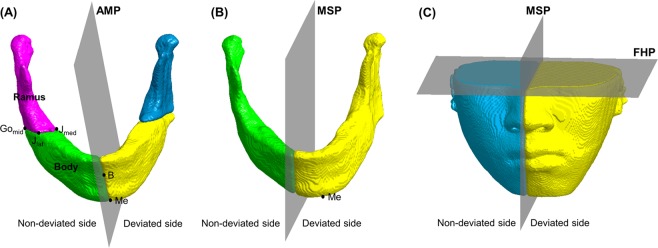
Segmentation of the mandible and lower facial soft tissue. (**A**) Mandible segmentation with absolute mandibular midsagittal plane (AMP) as the reference plane; (**B**) Mandible segmentation with facial midsagittal plane (MSP) as the reference plane; (**C**) Lower facial soft tissue segmentation with MSP as the reference plane. Me, menton; B, supramentale; Go_mid_, gonion midpoint; FHP, Frankfort horizontal plane.

The ramal length was defined as the distance between Con_sup_ and Go_mid_, and the body length was defined as the distance between Go_mid_ and Me^12,13^. The mandible was divided into two hemi-mandibles by the plane connecting Me, B, and G, which was defined as the AMP (Fig. 1). Hemi-mandibles were then divided into ramus and body by the plane connecting Go_mid_, J_lat_, and J_med_. To divide the mandible into ramus and body, then extract the corresponding data, we established a reference plane that could anatomically divide both sides of the mandible, rather than the MSP; based on previous studies, the AMP was used in this manner^12,13^. The mandible was also divided into two halves by using the MSP. In the conventional method for asymmetry evaluation, the surface area and volume measurements of the hemi-mandibles and 2 bony segments (ramus and body) on the Dev and N-Dev sides at T1 and T2 were obtained and differences in bilateral measurements were calculated. The surface area was calculated using distance around the boundary multiplied by the square of voxel size. The volume was calculated using the number of voxels in each image multiplied by the cube of voxel size. These data were extracted using MATLAB 2018a, in the same manner as that involving the use of Invivo 6 for evaluation of mandibular asymmetry in previous studies^29^.

#### Lower facial soft tissue measurements and bilateral differences

The volumes above the FHP were removed from soft tissue images. Then, images were divided into two halves by using the MSP (Fig. 1). The surface area and volume measurements of each hemi-lower face at T1 and T2 were obtained and differences in bilateral measurements were calculated. The surface area on the cutting plane was excluded from measurement. These data were extracted using MATLAB 2018a, in the same manner as that involving the use of Invivo 6 for evaluation of mandibular asymmetry in previous studies^29^.

#### Non-overlapping volume and similarity index by mirroring technique

**Figure 2:**
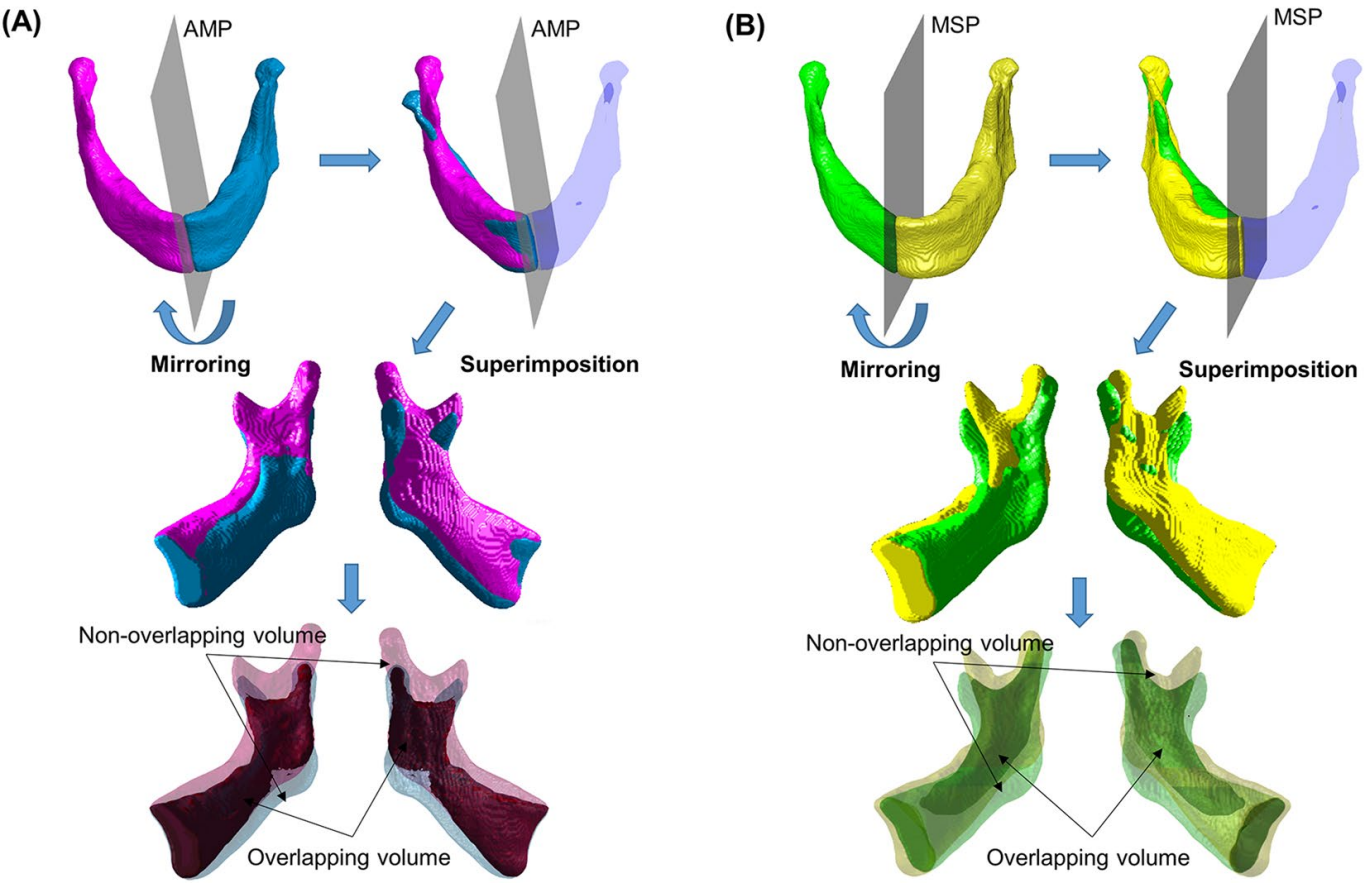
Procedure of mirroring and superimposition of the mandible and non-overlapping volume. (**A**) With absolute mandibular midsagittal plane (AMP) as the reference plane; (**B**) With facial midsagittal plane (MSP) as the reference plane. The opaque section in the figure indicates overlapping volume and the translucent section indicates non-overlapping volume.

The mandible was divided into two halves by using AMP or MSP. The mirror image of the left hemi-mandible was created by reflection along the reference planes and was superimposed on the right hemi-mandible (Fig. 2). When the two images were overlapped, the volume which was not shared between images, and which protruded from the overlapped image, was defined as non-overlapping volume. The 3D similarity index (Sørensen-Dice similarity coefficient) was obtained using the below equation, in order to evaluate the ratio of overlapping volume to whole mandibular volume; this indicates the degree of mandibular symmetry. The similarity index has a value from 0 to 1; values closer to 1, indicate that the bilateral mandibular structure is more symmetrical to the reference plane. The lower facial soft tissue was also mirrored and superimposed along the MSP in the same manner; the non-overlapping volume and similarity index parameters were calculated.

$${\rm{Similarity}}\,{\rm{index}}=2\ast |{\rm{intersection}}({\rm{A}},{\rm{B}})|/(|{\rm{A}}|+|{\rm{B}}|)$$

**Figure 3:**
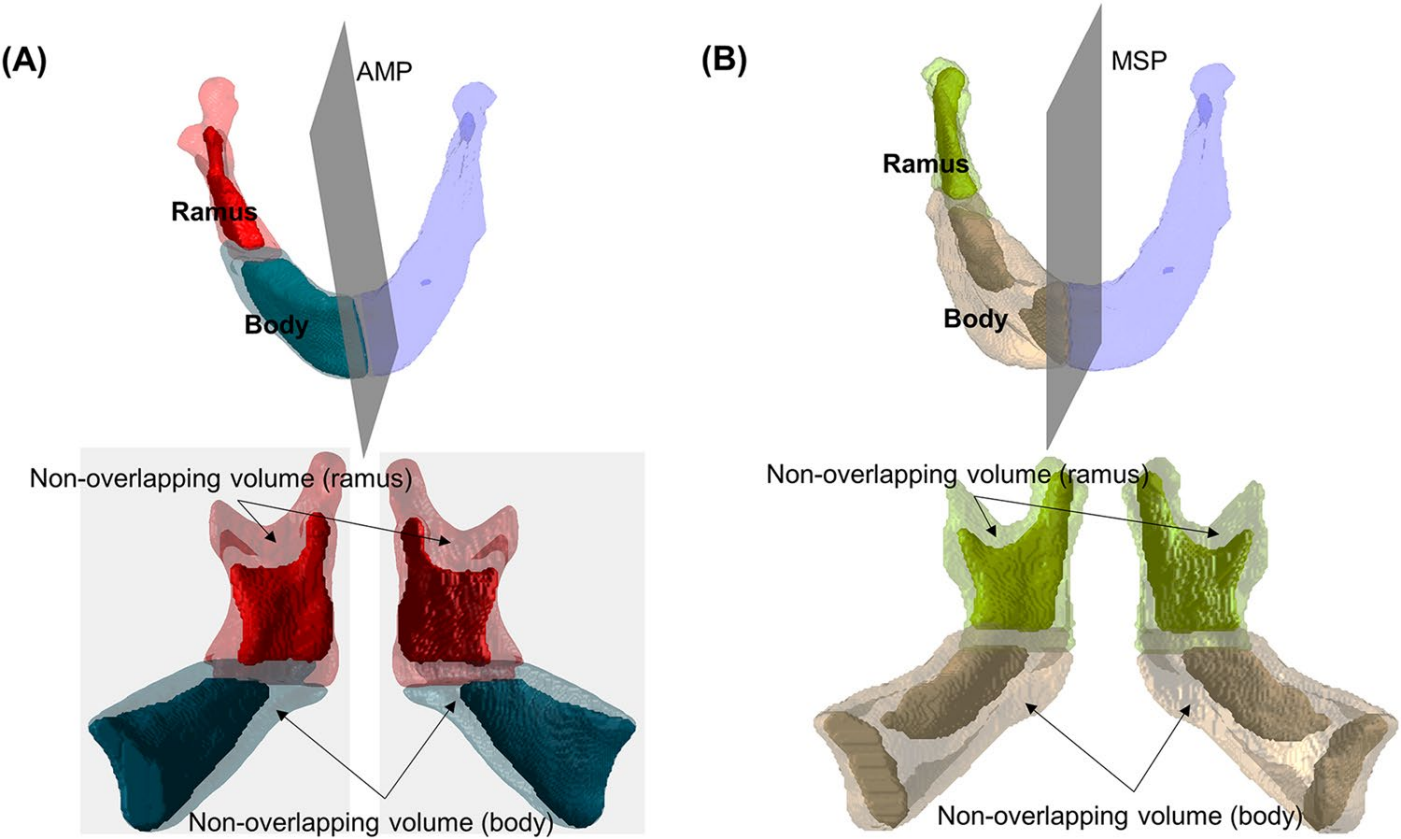
Segmentation of the hemi-mandible into ramus and body segments after mirroring and superimposition procedure. (**A**) With absolute mandibular midsagittal plane (AMP) as the reference plane; (**B**) With facial midsagittal plane (MSP) as the reference plane. The opaque section in the figure indicates overlapping volume and the translucent section indicates non-overlapping volume.

The distribution of non-overlapping volume after mirroring and superimposition was examined by dividing the hemi-mandible into ramus and body segment at T1 and T2 (Fig. 3). The mirroring technique in this study is based on the approach used in previous studies^18,19^, and all data related to the non-overlapping volume and similarity index were extracted using MATLAB 2018a by applying a customised code based on the above formula.

#### Reliability

In order to confirm reproducibility, a second set of measurements were made in 20 randomly selected subjects, 2 weeks after the first measurements, by a single investigator. The intraclass correlation coefficients ranged from 0.994 to 0.999.

#### Statistical analysis

SPSS software for Windows, version 21.0 (IBM Corp., Armonk, NY, USA), was used for statistical analyses. The minimum sample size was calculated based on a preliminary study for detection of the statistical significance of the change in the similarity index at each time point, using G*Power 3 (Düsseldorf, Germany) with a significance level of *P* < 0.05, a power of 80%, and an effect size of 0.5; this sample size was 34. The Shapiro-Wilk test was used to confirm normality of the data. Paired *t* tests were used to detect statistical significance of the difference in bilateral mandibular and soft tissue symmetry measurements at each time point, as well as changes in bilateral measurement difference, non-overlapping volume, and similarity index of the mandible and hemi-lower face over time. Additionally, we analysed correlations between the similarity index and non-overlapping volume, and between the similarity index and bilateral measurement difference, using Pearson’s correlation analysis. *P* values less than 0.05 were considered to be statistically significant.
